# Supplementary material for: Serum Level of Cytokeratin 18 (M65) as a Prognostic Marker of High Cardiovascular Disease Risk in Individuals with Non-Alcoholic Fatty Liver Disease
Source: Biomolecules. 2023 Jul 14;13(7):1128. doi: 10.3390/biom13071128 (PMC10377236; doi:10.3390/biom13071128)
Supplement: Supplementary file 1 [file biomolecules-13-01128-s001.zip › biomolecules-2482673-supplementary.pdf]

Supplemental tables

Table S1. Performance of CK18 (categorical or continuous value) in predicting FLI≥60 in PREVEND participants.

	All PREVEND participants (n=312)								
	**Discriminant accuracy**			**Univariate analysis**			**Multivariate analysis**		
**Predictor**	**AUC**	**95% CI**	***P*-value**	**OR**	**95% CI**	***P*-value**	**OR**	**95% CI**	***P*-value**
M30	0.702	0.641-0.762	<0.0001	1.004	1.002-1.006	0.0002	1.002	0.99-1.004	0.10
M65	0.657	0.59-0.719	<0.0001	1.004	1.002-1.006	<0.0001	1.003	1.000-1.005	0.004
M30>200	-	-	-	3.42	2.11-5.56	<0.0001	3.22	1.92-5.39	<0.0001
M65>400	-	-	-	2.61	1.18-5.75	0.01	1.35	0.58-3.14	0.48

AUC: area under the curve; OR: odds ratio; CI: confidence interval; CK: Cytokeratin.
AUC non calculated for CK18 categorical values. In multivariate analysis, only M30 M65 were considered in the model.

Table S2. *P*-value for Spearman's rank correlation between levels of CK18 (M30 and M65) and clinical characteristics of PREVEND participants.

	**All PREVEND participants (n=312)**		**FLI≥60 PREVEND participants (n=112)**		**FLI<60 PREVEND participants (n=200)**	
	**M30**	**M65**	**M30**	**M65**	**M30**	**M65**
**Variable**	***P-value***	***P-value***	***P-value***	***P-value***	***P-value***	***P-value***
Age	<0.0001	<0.0001	0.15	0.006	0.03	0.009
Waist circumference	<0.0001	<0.0001	0.89	0.87	0.01	0.30
Weight	<0.0001	0.01	0.08	0.09	0.18	0.37
BMI	<0.0001	0.008	0.38	0.51	0.66	0.57
DBP	0.01	0.36	0.43	0.26	0.54	0.92
SBP	<0.0001	0.0004	0.01	0.03	0.02	0.22
Total -c	0.06	0.04	0.001	0.001	0.97	0.98
LDL-c	0.01	0.02	<0.0001	0.001	0.89	0.94
HDL-c	0.002	0.01	0.74	0.83	0.50	0.88
TG	<0.0001	0.003	0.6	0.75	0.14	0.42
Plasma glucose	<0.0001	<0.0001	0.02	0.005	0.02	0.002
ALP	0.001	0.01	0.03	0.09	0.15	0.19
ALT	0.0001	0.0004	0.0008	0.003	0.44	0.37
AST	<0.0001	<0.0001	<0.0001	<0.0001	0.0005	0.001
GGT	<0.0001	<0.0001	<0.0001	0.0006	0.003	0.0003
FLI	<0.0001	<0.0001	0.05	0.14	0.004	0.09
FRS	0.0001	0.0006	0.17	0.03	0.15	0.23

SCORE2	0.0003	0.0001	0.24	0.05	0.10	0.07

Abbreviations: Total-C: total cholesterol; LDL-C: low-density lipoprotein cholesterol; HDL-C: high-density lipoprotein cholesterol ; TG: triglycerides; BMI: body mass index; DBP: Diastolic blood pressure; SBP: Systolic blood pressure ALT: alanine transaminase; AST: aspartate transaminase; FLI: fatty liver index; ALP: alkaline phosphatase; GGT: gamma-glutamyl-transferase; FRS: Framingham risk score; SCORE: Systematic COronary Risk Evaluation.
